# Optical Coherence Tomography (Angiography) Biomarkers in the Assessment and Monitoring of Diabetic Macular Edema

**DOI:** 10.1155/2020/6655021

**Published:** 2020-12-31

**Authors:** Corina-Iuliana Suciu, Vlad-Ioan Suciu, Simona-Delia Nicoara

**Affiliations:** ^1^Medical Doctoral School, University of Oradea, Oradea 410087, Romania; ^2^Medical Doctoral School, “Iuliu Hațieganu” University of Medicine and Pharmacy, Cluj-Napoca 400012, Romania; ^3^Department of Ophthalmology, “Iuliu Hațieganu” University of Medicine and Pharmacy, Cluj-Napoca 400012, Romania; ^4^Emergency County Hospital, Cluj-Napoca 400006, Romania

## Abstract

Retinopathy is one of the most severe diabetes-related complications, and macular edema is the major cause of central vision loss in patients with diabetes mellitus. Significant progress has been made in recent years in optical coherence tomography and angiography technology. At the same time, various parameters have been attributed the role of biomarkers creating the frame for new monitoring and treatment strategies and offering new insights into the pathogenesis of diabetic retinopathy and diabetic macular edema. In this review, we gathered the results of studies that investigated various specific OCT (angiography) parameters in diabetic macular edema, such as central subfoveal thickness (CST), cube average thickness (CAT), cube volume (CV), choroidal thickness (CT), retinal nerve fiber layer (RNFL), retinal thickness at the fovea (RTF), subfoveal choroidal thickness (SFCT), central macular thickness (CMT), choroidal vascularity index (CVI), total macular volume (TMV), central choroid thickness (CCT), photoreceptor outer segment (PROS), perfused capillary density (PCD), foveal avascular zone (FAZ), subfoveal neuroretinal detachment (SND), hyperreflective foci (HF), disorganization of the inner retinal layers (DRIL), ellipsoid zone (EZ), inner segment/outer segment (IS/OS) junctions, vascular density (VD), deep capillary plexus (DCP), and superficial capillary plexus (SCP), in order to provide a synthesis of biomarkers that are currently used for the early diagnosis, assessment, monitoring, and outlining of prognosis.

## 1. Introduction

Diabetic retinopathy (DR) is the leading cause of blindness in people under 75 years of age in developed countries [1, 2]. Diabetic macular edema (DME) can occur at any stage of DR, being the major cause of central vision loss in patients with diabetes mellitus (DM) [3]. The World Health Organization estimated that by the year 2030, there will be approximately 366 million individuals suffering from DM [4]. Therefore, the study of DME with the aim to prevent vision loss is of utmost importance. The understanding and characterization of DME are essential for its prevention and for the development of new targeted treatments [5].

The transparency of ocular structures and the examining of living retina offer valuable insights into the microvascular changes subsequent to long-term exposure to hyperglycemia in patients with DM [6]. Optical coherence tomography (OCT) provides cross-sectional images of the retinal microstructures being able to measure the retinal thickness (RT) and identify DME before its clinical appearance [5]. Parallel with the development of OCT technology, various parameters have been attributed the role of biomarkers creating the frame for new monitoring and treatment strategies and offering new insights into the pathogenesis of DR. Although the pathogenesis of DME is focused mainly on the breakdown of the inner blood-retina barrier (BRB), improvements in the visualization of the choroid by enhanced depth imaging-optical coherence tomography (EDI-OCT) and swept source-optical coherence tomography (SS-OCT) set the stage for investigating choroidal biomarkers in patients with DME.

OCT angiography (OCTA) is a noninvasive technique that allows to visualize the retinal plexuses layer by layer, to quantify microvascular parameters, and to correlate them with functional and morphological data [7].

Research is oriented towards identifying earlier preclinical biomarkers of microvascular abnormality in diabetic retina which is very important considering that early treatment is associated with better outcome [6]. Novel preclinical biomarkers could also draw attention on the pathogenesis of DR.

In this narrative review, we gathered the results of studies that investigated specific parameters in DME using OCT and OCTA in order to provide a synthesis of biomarkers that are currently used to assess, monitor, and outline the prognosis of this condition.

## 2. Development

Currently, OCT is an invaluable and indispensable tool for the monitoring of patients with diabetes, establishes the need for treatment, and formulates prognosis [8].

### 2.1. Macular Thickness and Volume

Diabetic macular edema (DME) is identified by the thickening of the retina as a result of excessive fluid accumulation [5] caused by the breakdown of (BRB) [8]. The fluid may be extracellular, intracellular, or mixed. Santos et al. aimed to characterize the type of retinal edema in the initial stages of retinopathy in patients with type 2 diabetes mellitus (T2DM) [9]. The authors used the classification proposed by Klatzo to characterize the macular edema as cytotoxic (intracellular) or vasogenic (extracellular) [10]. They showed that in the initial stages, the edema was predominantly intracellular, as a result of cytotoxic damage of the Müller cells and of other neuronal cells. As the disease progressed, the breakdown of the BRB predominated with resulting extracellular (vasculogenic) edema [5]. According to their study, macular edema occurred independently of the severity of DR. The authors found that the inner nuclear layer showed a higher and most frequent increase in retinal thickness (RT) [5]. By multimodal imaging of the initial stages of DR, the same authors found that the eyes with DME from different patients included in the same Early Treatment Diabetic Retinopathy Study (ETDRS) grading category displayed different prevalence of the main disease pathways, neurodegeneration, edema, and ischemia [9, 11]. This observation supports the theory of different phenotypes of disease progression.

In 2019, Saxena et al. suggested that three OCT biomarkers proved their validity in DME as diagnostic and predictive factors: mean central subfield thickness (CST), cube average thickness (CAT), and cube volume (CV) [12]. CST is defined as the thickness of a central circle of 1 mm diameter in the circular ETDRS grid map. CAT represents the overall average thickness of the tissue layers between internal limiting membrane and retinal pigmented epithelium (ILM-RPE) over the entire 6 × 6 mm square scanned area, the mean of thicknesses in nine sections. CV is defined as the overall average volume of the tissue layers between ILM-RPE over the entire 6 × 6 mm square scanned area. The authors revealed a statistically significant difference in CST, CAT, CV, and logMAR visual acuity between cases with DME and cases without DME, regardless the staging of DR. They concluded that CST, CAT, and CV are independent markers of severity of retinopathy and predictors of visual acuity [12].

When edema overcomes the stretching capability of the retina, bipolar axons are damaged with subsequent disruption of visual signal transmission. As a consequence of these morphological changes, the recovery of visual acuity does not parallel the resolution of edema. Therefore, according to another report, CST is not a reliable biomarker to evaluate the prognostic in patients with DME and the attention must be directed to examining the pattern of edema, its extent, and location relative to the inner and outer retina [8]. Pelosini et al. proved that the cross-sectional area between the retinal plexiform layers is a better predictor of visual acuity than macular thickness [13].

### 2.2. Subfoveal Neurosensory Detachment

**Figure 1:**
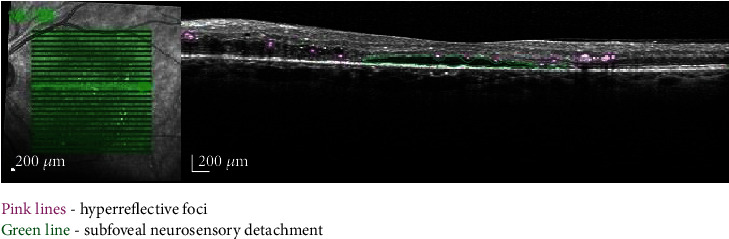
Macular OCT image revealing SND and HF.

DME can have various aspects on OCT: sponge-like swelling, cystoid macular edema, and subfoveal neuroretinal detachment (SND). The latter one has a reported prevalence of 15–30% in eyes with DME, and it appears on OCT as a hyporeflective area beneath the neuroretina [14] (Figure 1). Various hypotheses have been advanced regarding the pathogenesis of SND. The main mechanism is considered to be the leakage from the retinal or choroidal circulation into the subretinal space exceeding the reabsorption capacity [14]. In diabetic retinopathy, the RPE is altered [15] or its capacity reduced because of local hypoxia [16]. The condition of external limiting membrane (ELM) seems to be important for the pathophysiology of SND. In eyes with DME, there is a breakdown of inner BRB which causes extravasation of lipids and proteins, but as long as ELM is intact, they accumulate anterior to it causing the swelling of the outer retina. When ELM is compromised, proteins and fluid may move through it into the subretinal space, resulting in the development of SND. The study proved that the disruption of ELM correlates with the presence and height of SND in eyes with DME [14]. Vujosevic et al. found that DME with SND correlates with greater choroidal thickness (CT), more hyperreflective foci (HF), disruption of the ELM, and significant impairment of the macular function translated by the decrease of retinal sensitivity (RS) [14]. In SND+ eyes, an inverse correlation was identified between CT and RS, and in SND- eyes, a direct correlation between CT and RS was found, suggesting that DME with SND+ and SND- are two different morphologic and functional entities [14]. Functional impairment in eyes with SND+ and SND- indicates the importance of the choroid for RS [14].

On the other hand, many studies demonstrated the protective effect of SND in the sense that its presence was associated with better visual gains at the end of one year, including in the subgroup of patients with pars plana vitrectomy for diffuse DME [17]. It was also reported that SND at baseline was associated with better response to intravitreal aflibercept [18] and dexamethasone implants [19].

SND is an important OCT biomarker, but its role as an anatomic and functional prognostic factor needs further investigation [8].

### 2.3. Intraretinal Cystoid Spaces

**Figure 2:**
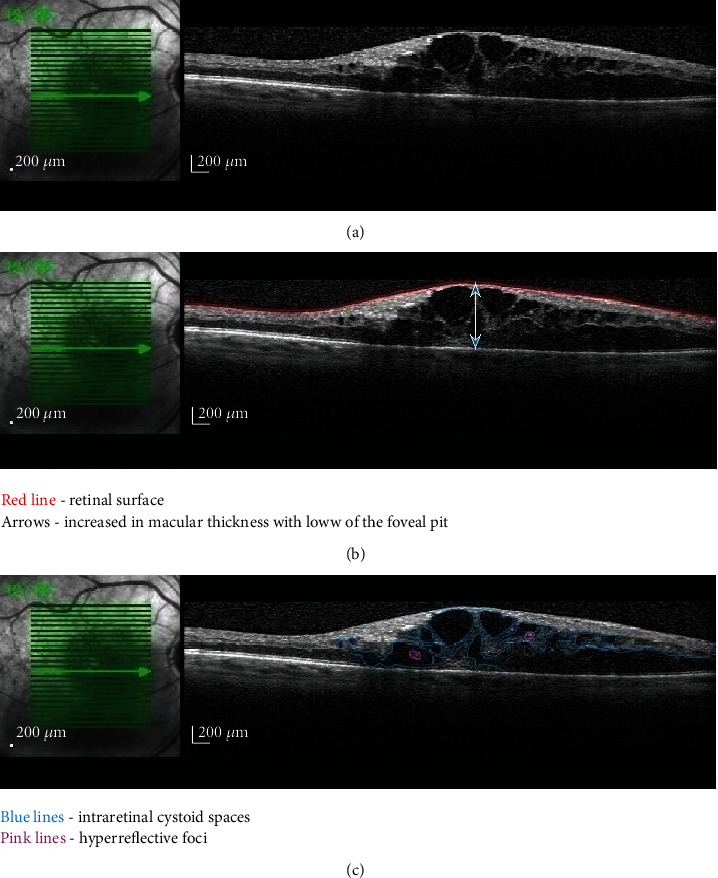
OCT aspects of the intraretinal cystoid spaces: (a) original OCT image; (b, c) highlighted lesions of the same image.

The formation of intraretinal cysts is the consequence of inner BRB disruption in diabetes as a consequence of elevated VEGF levels [8]. Cystic spaces within the macula (Figure 2) are the expression of coalescent extracellular fluid resulting from the malfunctioning of Müller cells that act like pumps to keep the macula dry [20]. The prognostic significance of intraretinal cystoid spaces depends on their size, location, and association of hyperreflective material. Based on their size, the cysts were classified as small (<100 *μ*m), large (101-200 *μ*m), and giant (>200 *μ*m). The larger size of the cysts is associated with macular ischemia, being poor prognostic factors for visual acuity. Large and giant intraretinal cysts affect the outer nuclear layer (ONL) and damage the IS/OS junction with irreversible loss of the visual function [21]. The hyperreflective material forms septa within the cysts; it is hypothesized to be fibrin and inflammatory by-products and signifies the severe disruption of BRB, being associated with poor outcome of visual acuity following treatment with anti-VEGF agents [20, 22]. Al Faran et al. identified that bridging between the cystic cavities is associated with better functional outcomes following bevacizumab injections as opposed to its absence [23]. The bridging tissue represents residual neuronal material connecting the outer and inner retina with subsequent improvement of transmitting visual impulses to the optic nerve axons. If the bridging process does not occur, the outcome is poor with resulting retinal thinning and atrophy [23].

### 2.4. HF

**Figure 3:**
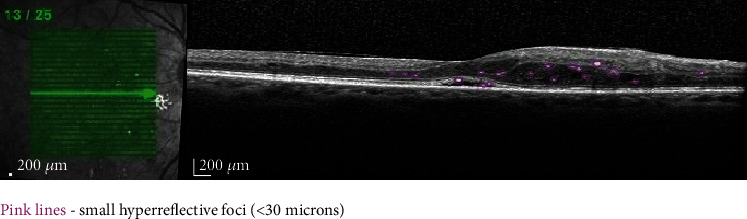
Highlighted OCT image revealing the small HF.

Vujosevic et al. described three types of HF according to their appearance and location, with various meanings: ≤30 *μ*m diameter, reflectivity similar to nerve fiber layer, absence of back shadowing, and location in the inner and outer retina may be associated with activated microglial cells (Figure 3); >30 *μ*m diameter, reflectivity similar to EPR-Bruch membrane complex, presence of back shadowing, and location in the outer retina may represent hard exudates; and >30 *μ*m diameter, reflectivity similar to EPR-Bruch membrane complex, the presence of back shadowing, and location in the inner retina may represent microaneurysms [24]. Small HF (≤30 microns) are proposed as imaging OCT biomarkers of retinal inflammation in eyes with DME [14]. They were postulated to be fine lipid or protein deposits originating in the breakdown of BRB and anticipating the appearance of hard exudates [25, 26]. According to other theories, HF result from a neurodegenerative process and they precede the development of DR [27]. A significant correlation was found between the number of small HF and the presence of SND supporting the theory of a major inflammatory condition in this pattern of DME [14].

In 2019, Liu and colleagues evaluated the role of OCT in predicting the response to anti-VEGF treatment in DME. They used conbercept (KH902; Chengdu Kanghong Biotech Co., Ltd., Sichuan, China), a new anti-VEGF drug similar to aflibercept, binding to VEGF receptors 1 and 2, which has been demonstrated to be effective in treating DME [28, 29]. When compared to ranibizumab in the treatment of DME, conbercept achieved similar clinical efficacy with longer treatment intervals and fewer intravitreal injections [29]. The authors noticed a reduction in the number of HF on the OCT scans following the administration of conbercept, asserting that HF on OCT scans are reliable biomarkers of individual response to conbercept treatment in patients with DME. A greater number of HF on the OCT scans at baseline demonstrates a more active DME and predicts worse final best-corrected visual acuity following conbercept treatment [3].

HF larger than 30 *μ*m and with back shadowing, located in the outer retina, are suggestive for hard exudates, meaning lipoprotein deposits due to BRB breakdown. It was proved that they are associated with serum lipid levels and that elevated triglyceride levels are associated with subfoveal location of hard exudates [30]. If they are located subfoveally, intravitreal implants with steroids may be more effective than anti-VEGF agents [31]. The OCT monitoring of hard exudates could be useful in assessing the response to treatment of DME [32].

### 2.5. Disorganization of the Inner Retinal Layers (DRIL)

**Figure 4:**
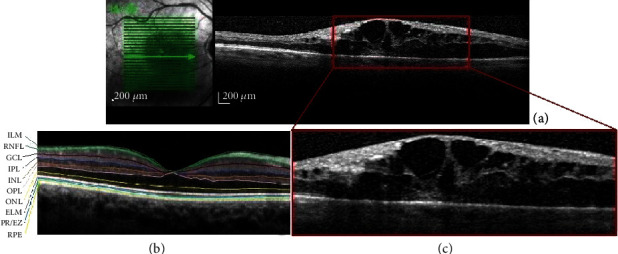
OCT aspect of macular DRIL. (a) Disorganization of the inner retinal layers (DRIL). (b) Normal macular segmentation. (c) Magnified segment of the first image (a); global disruption of the ellipsoid zone and RNFL. ILM: internal limiting membrane; RNFL: retinal nerve fiber; GCL: ganglion cell layer; IPL: inner plexiform layer; INL: inner nuclear layer; OPL: outer plexiform layer; ONL: outer nuclear layer; ELM: external limiting membrane; PR/EZ: photoreceptor layer/ellipsoid zone (inner and outer photoreceptor segment junction); RPE: retinal pigment epithelium.

DRIL is a novel and recently described biomarker which is not specific to DR but develops in multiple retinal diseases as a response to retinal stress [20, 33]. DRIL signifies the poor definition of the boundaries of the inner retinal layers [33] (Figure 4).

Nadri et al. were the first to study the correlation between DRIL, macular thickness parameters, disruption of the ellipsoid zone (EZ), and retinal nerve fiber layer (RNFL) thickness in DR using Spectral Domain OCT (SD-OCT) [34]. DRIL was graded as 0 (absent) or 1 (present). EZ was graded as intact (grade 0), with focal disruption (grade 1) and with global disruption (grade 2). DRIL was significantly associated with the severity of DR. There was a significant positive correlation between DRIL and CST, CAT, and the grades of EZ disruption and a significant negative correlation between DRIL and RNFL thickness [34].

Das et al. formulated the question of whether retinal morphology evaluated by SD-OCT can be a potential biomarker in eyes with DME [20]. They found that DRIL was identified more frequently in eyes with increasing severity of DR and it was associated with worse outcomes of visual acuity. The authors also noted that for each 100 *μ*m horizontal increase of DRIL, there was a negative impact on visual acuity of more than one line on the ETDRS chart [20]. One possible explanation is given by the mechanical theory according to which bipolar axons snap when their elasticity limit is exceeded by edema, leading to the disorganization of the inner retina [13]. DRIL was significantly associated with disruption of the outer retinal layers (ELM and EZ) and with the increase of the retinal thickness at the fovea (RTF) [20]. When analyzed together, these findings suggest that the same mechanisms are responsible for the disorganization of the inner retina and for the disruption of the outer retina [20]. Currently, it is not known whether the EZ line corresponds histologically to the junction of the inner and outer segments. This study underlines the clinical significance of an intact EZ and suggests that DRIL and the disruption of the outer retina have the same pathogenic mechanisms. Since DRIL correlated significantly with the severity of DR, the authors assume that the finding of worse visual acuities associated with more severe DR may be the consequence of DRIL [20].

Jolitkov et al. set the objective to elucidate the relationship between DRIL and the retinal function in patients with diabetes without DR and with nonproliferative diabetic retinopathy (NPDR) but without DME [33]. DRIL was identified in SD-OCT scans in 16% of patients with diabetes and in none of the controls. In addition to ETDRS visual acuity testing, the authors used an automated contrast sensitivity method and three visual field testing strategies [33]. DRIL was associated with a measurable degree of retinal dysfunction, even if the neuroretinal impairment was in an early stage [33]. When comparing DRIL with OCT thickness, the authors found that DRIL was associated with retinal thinning mostly in the inner retina but also in the outer retina [33]. The likelihood of DRIL was greater in mild to moderate NPDR as compared to patients with diabetes without retinopathy. The study also found that the patients with DRIL had higher body mass index and longer duration of DM. The findings of this study highlight the correlation between retinal structure and function, and it confers DRIL the status of a reliable and readily available biomarker to monitor the neuroretinal impairment in DM [33].

### 2.6. Vitreomacular Interface

**Figure 5:**
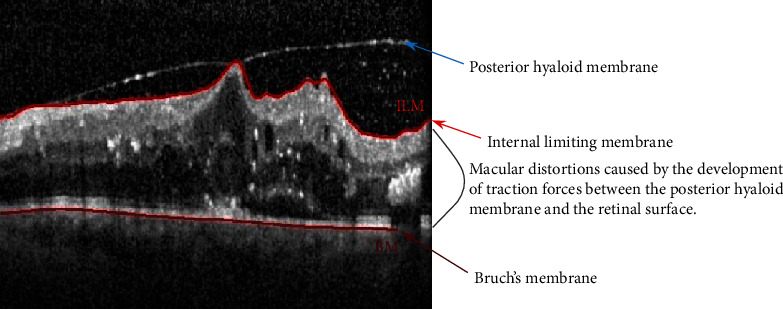
OCT aspects of the vitreomacular interface showing taut posterior hyaloid membrane with subsequent macular distortion.

In patients with diabetes mellitus, often the posterior hyaloid forms a sheet along the posterior pole with the subsequent development of traction forces and macular distortion (Figure 5). The term describing this abnormal vitreomacular relationship is taut posterior hyaloid membrane and is responsible for recalcitrant macular edema. OCT reveals taut posterior hyaloid membrane, identifying the patients with DME who could benefit from pars plana vitrectomy and removal of the posterior hyaloid [35].

### 2.7. Outer Retina

**Figure 6:**
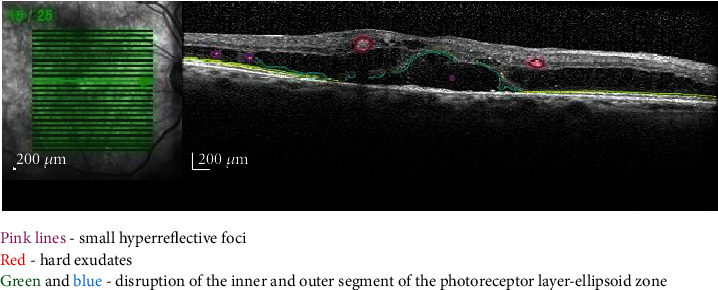
Highlighted OCT image showing HF, hard exudates, and the disruption of IS/OS photoreceptor segments (EZ).

The OCT imaging of the outer retinal layers offers valuable information on the health of photoreceptors and RPE (Figure 6). Zur et al. described three grades of IS/OS junction aspects: continuous, partly disrupted, and completely disrupted, and concluded that eyes with intact IS/OS junctions have better outcomes following treatment with dexamethasone implant [36]. Ota et al. found that visual acuity is positively correlated with the survival rate of ELM and with the EZ which are affected by long-term DME [37].

Photoreceptor outer segment (PROS) is defined on OCT as the distance between IS/OS junction and RPE. There is evidence that shorter PROS were significantly associated with the presence of DR or DME [38] and with worse visual acuity in patients with DME [39].

### 2.8. Choroidal Biomarkers

The choroid provides the blood supply to the RPE and photoreceptor cells, playing a major role in the metabolic exchange to the foveal avascular zone (FAZ). Endo et al. determined the central choroid thickness (CCT) based on EDI-OCT in patients with treatment naïve DME in comparison to patients with diabetes without DME. CCT layer was significantly thicker in patients with treatment naïve DME as compared to patients without DME [40]. The authors selected untreated DME in order to eliminate the influence of various treatment modalities on CCT. Thus, panretinal laser photocoagulation [41], intravitreal anti-VEGF administration [42], and intravitreal triamcinolone acetonide injection [43] could affect CCT. Other studies found that the central choroid in patients with treatment naïve DME was thinned [44–47], thickened [48, 49], or unchanged [50–52]. The explanations of these conflicting results are various: different inclusion criteria regarding the staging of DR, small number of cases, patient background, and differences between races [40].

A study conducted by Sala-Puigdollers et al. evaluated the reliability of the next generation of OCT devices, the SS-OCT in DME [53]. SS-OCT operates up to 100.000 A-line scans per second and uses a laser source of a longer wavelength (1050 nm) that penetrates deeper in the retina and choroid than the conventional laser sources of the SD-OCT devices. The authors found good reliability, repeatability, and reproducibility of SS-OCT in quantifying retinal and choroidal thickness in DME cases [53]. Moreover, the authors claim that SS-OCT may become the gold standard technique for the evaluation of DME [53]. The studies that investigated the reproducibility of choroidal thickness measurements concluded that there is a low variability of this parameter acquired with SD-OCT and SS-OCT [54].

Abadia et al. compared the choroidal thickness between patients with T2DM and healthy age-matched controls using SS-OCT and found that overall, the patients with T2DM had thinner choroids than the normal controls [54]. All the measurements were performed within the same range of time during the day to avoid fluctuations due to the diurnal variations in choroidal thickness. An interesting observation was that in both groups, the choroid thickness had similar patterns: it was thickest in the subfoveal (SF) area followed by the temporal and nasal zones close to the SF area; the choroid was thinner in the temporal area far from the SF zone and thinnest nasally to the optic disc [54]. According to the same authors, within the group of patients with T2DM, the presence of DME did not influence the choroid thickness. However, the choroid was significantly thinner in patients with DME versus healthy controls, with the most important difference at the SF area. Currently, it is not known whether the thinning of the choroid is prior to the DR lesions or the DR structural changes result in the reduction of the choroidal thickness [54].

The choroidal vascularity index (CVI) is a novel OCT parameter for measuring the vasculature status of the choroid [55]. The CVI is a term introduced by Agrawal et al. and represents the ratio of choroidal luminal area to total choroidal area [56]. The CVI was recently introduced as a novel biomarker to monitor the progression of DR. Studies proved that while choroidal thickness is unaltered in DR, the CVI correlates with progressing DR [46]. More than that, the CVI is altered before the onset of DR, supporting the theory of choroidal primary damage in DR [8].

Using an EDI SD-OCT device, Gupta et al. evaluated the structural changes of the choroid in eyes with treatment naïve DME and various grades of DR versus healthy controls [57]. Gupta et al. found that the CVI was highest in patients with mild DR and lowest in patients with proliferative diabetic retinopathy (PDR), with a statistically significant difference across the DR severities and the control group. DME did not correlate significantly with the CVI. Subfoveal choroidal thickness (SFCT) increased with the severity of DR, but not in a statistically significant manner. SFCT had a positive significant correlation with the central macular thickness (CMT) and total macular volume (TMV). A negative correlation, although insignificant, was found between SFCT and CVI. This study concludes that the CVI has the potential to be useful in monitoring the progression of DR and DME and offers an additional insight in elucidating the pathogenesis of the disease by tracking the structural changes in the choroid [57].

According to a study conducted by Rayess et al., SFCT is a predictor of response to anti-VEGF therapy [42]. The authors found that a greater SFCT at baseline is associated with better outcomes following anti-VEGF treatment. The possible explanation is that greater choroidal thickness is associated with intact choriocapillaris, less ischemic outer retina, and better preservation of photoreceptors [42].

Hyperreflective choroidal foci (HCF) were described recently, and they represent lipofuscin deposition in the choroidal layers [58]. HCF signal poor prognostic for visual acuity, their number being significantly higher in eyes with PDR versus NPDR [8].

### 2.9. OCTA Biomarkers

OCTA makes it possible to visualize the retinal vascular plexuses, which is impossible with fluorescein angiography [7].

AttaAllah et al. aimed to evaluate macular perfusion using OCTA automated software algorithms in patients with treatment naïve DME and moderate to severe NPDR [59]. The macular area vascular density (VD) and FAZ were assessed and compared between three groups: diabetic eyes with DME, diabetic eyes without DME, and healthy controls. The authors found that eyes with DME had significantly lower vessel densities at the level of deep capillary plexus (DCP) and FAZ was significantly larger at the level of the superficial capillary plexus (SCP) when compared with diabetic eyes without macular edema and controls. In patients with DME, eyes with larger FAZ had worse visual acuity. The authors conclude that the above-mentioned OCTA biomarkers could be used to predict the evolution of visual acuity and to monitor the response to treatment [59].

Parravano et al. combined OCT and OCTA parameters to investigate the progression of diabetic microaneurisms (MA) and to quantify their effect on the accumulation of retinal extracellular fluid at 1 year follow-up in patients with NPDR [60]. The following MA parameters were evaluated by SD-OCT: the visibility, the changes of internal reflectivity (graded as hyporeflective, moderate, or hyperreflective), and the amount of fluid surrounding each MA. The changes in the visualization of SCP and DCP and the flow in the corresponding OCTA scans were evaluated. The extracellular fluid accumulation at 1 year was strongly associated with the reflectivity pattern of the MAs at baseline, with hyperreflective MAs being significantly associated with an increased risk of fluid accumulation as compared to the hyporeflective ones. The development of extracellular fluid at 1 year was significantly associated with the presence of flow, the visibility, and the deep location of MAs [60]. The authors conclude that OCT and OCTA parameters of MAs predict the retinal extracellular fluid accumulation at one year in patients with NPDR; therefore, a better interpretation of MAs could improve the timing of treatment in DME [60].

Tang et al. focused on the investigation of OCTA parameters related to the DCP in patients with DM included in one of the following categories: without DR, mild DR, moderate DR, or severe DR [7]. Three parameters were calculated: FAZ, vascular density (VD), and fractal dimension (FD). Larger FAZ was associated with more severe DR, shorter axial length (AL), thinner SFCT, and lower body mass index (BMI) [7]. Lower VD was associated with more severe DR, shorter AL, and worse visual acuity. Lower FD was associated with more severe DR and older age [7]. The authors concluded that the effect of ocular and systemic factors should be considered in order to interpret correctly OCT and OCTA parameters [7]. Decreased VD in the DCP was associated with worse visual acuity, suggesting that VD in DCP may reflect the degree of capillary loss in patients with visual deterioration related to DME [7]. DCP supplies 10 to 15% of the oxygen for the photoreceptors. Since DCP is the first affected in DM, OCTA evaluation could predict the evolution of visual acuity at an early stage, facilitating the monitoring and management of patients with DM [7]. The severity of DR was associated with all DCP metrics, but in a multivariable analysis, only the most severe category of DR was related to an increased FAZ due to the high variability of the FAZ itself among even normal individuals. The association of lower SFCT with more advanced stages of DR suggests that choroidal vascular abnormalities occur simultaneously with or as a result of DR [7]. Older age was related to reduced FD, since aging is associated with decreased complexity of organ structure [7]. Vascular structure changes in obesity, including thickened basement membranes, increased vascular diameter, and stiffened arterioles with reduced lumen size explain the OCTA findings associated with increased BMI, namely, increased FD and FAZ in DCP [7]. The increased diameter and thickening in case of increased BMI lead to an increased occupancy of vessels in OCTA images which translate into increased FD and decreased FAZ area. However, the underlying mechanism remains unclear, so these findings should be interpreted with caution.

Rosen et al. brought evidence of preclinical DR by comparing perfused capillary density (PCD) in patients with diabetes against healthy controls using OCTA [6]. The patients with diabetes without retinopathy demonstrated consistently higher PCD compared to the control group, reaching statistical significance. The NPDR and PDR groups showed progressively decreasing PCD. Regarding FAZ metrics, there was no statistically significant difference between the No DR group and controls [6]. Notably, PCD was more sensitive than FAZ metrics in detecting the differences between the No DR and control groups. Increased PCD values in the No DR group as compared to controls could be explained by autoregulation as a response to increased metabolic demand. The PCD decrease in the NPDR and PDR groups results from the incremental loss of capillary segments [6]. This shift of PCD from elevation to progressive loss marks the key moment of the compensatory response just preceding the appearance of clinical signs [6]. Therefore, the decline of PCD may have the value of a biomarker signaling the risk of visual loss and other systemic complications [6].

In a recent paper, Veiby et al. describe important findings in a cohort of patients with type 1 DM using OCTA [61]. The authors found that lower VD in DCP was the only OCTA factor associated with the progression of NPDR. Since the decrease of VD in DCP occurs before the presence of apparent retinopathy, it could be attributed the role of an early noninvasive biomarker for the progression of DR, being superior to OCT in detecting changes associated with NPDR progression without macular edema. According to the same study, the FAZ area measured by OCTA was not significantly associated with the NPDR level, but it was significantly higher in the severe NPDR group compared to other groups. Based on the results of their study, the authors suggest a new classification system of DR based on OCTA measurements [61].

## 3. Conclusions

Recent progress in OCT and OCTA imaging techniques led to the identification of new parameters having the potential of biomarkers in DME. The possibility to investigate the choroid with EDI-OCT and SS-OCT paved the way for the discovery of new biomarkers. OCTA makes it possible to investigate noninvasively and individually the retinal vascular layers, to delineate precisely the vascularized from the nonvascularized areas, and to calculate various vascular parameters. Analysis of newly discovered biomarkers and their connection with those already known offered new insights into the pathogenesis, early diagnosis, and monitoring of diabetic retinopathy and diabetic macular edema and opened new avenues of research.

Based on the data presented, in parallel with the widespread use of OCT and OCTA in the clinical practice, future screening of DR should include these examinations for the assessment of DME with subsequent earlier diagnosis and better outcomes.

## References

[1] Aiello L. P., Gardner T. W., King G. L. (1998). Diabetic retinopathy. *Diabetes Care*.

[2] Klein R., Moss S. E., Klein B. E., Davis M. D., DeMets D. L. (1989). The Wisconsin epidemiologic study of diabetic retinopathy: XI. The incidence of macular edema. *Ophthalmology*.

[3] Liu S., Wang D., Chen F., Zhang X. (2019). Hyperreflective foci in OCT image as a biomarker of poor prognosis in diabetic macular edema patients treating with conbercept in China. *BMC Ophthalmology*.

[4] Kwan C. C., Fawzi A. A. (2019). Imaging and biomarkers in diabetic macular edema and diabetic retinopathy. *Current Diabetes Reports*.

[5] Daruich A., Matet A., Moulin A. (2018). Mechanisms of macular edema: beyond the surface. *Progress in Retinal and Eye Research*.

[6] Rosen R. B., Andrade Romo J. S., Krawitz B. D. (2019). Earliest evidence of preclinical diabetic retinopathy revealed using optical coherence tomography angiography perfused capillary density. *American Journal of Ophthalmology*.

[7] Tang F. Y., Chan E. O., Sun Z. (2020). Clinically relevant factors associated with quantitative optical coherence tomography angiography metrics in deep capillary plexus in patients with diabetes. *Eye and Vision*.

[8] Markan A., Agarwal A., Arora A., Bazgain K., Rana V., Gupta V. (2020). Novel imaging biomarkers in diabetic retinopathy and diabetic macular edema. *Therapeutic Advances in Ophthalmology*.

[9] Santos A. (2019). Characterization of initial stages of diabetic macular edema. *Ophthalmic Research*.

[10] Klatzo I. (1967). Presidental address. Neuropathological aspects of brain edema. *Journal of Neuropathology and Experimental Neurology*.

[11] Marques I. P., Alves D., Santos T. (2019). Multimodal imaging of the initial stages of diabetic retinopathy: different disease pathways in different patients. *Diabetes*.

[12] Saxena S., Caprnda M., Ruia S., Prasad S. (2019). Spectral domain optical coherence tomography based imaging biomarkers for diabetic retinopathy. *Endocrine*.

[13] Pelosini L., Hull C. C., Boyce J. F., McHugh D., Stanford M. R., Marshall J. (2011). Optical coherence tomography may be used to predict visual acuity in patients with macular edema. *Investigative Opthalmology & Visual Science*.

[14] Vujosevic S., Torresin T., Berton M., Bini S., Convento E., Midena E. (2017). Diabetic macular edema with and without subfoveal neuroretinal detachment: two different morphologic and functional entities. *American Journal of Ophthalmology*.

[15] Weinberger D., Fink-Cohen S., Gaton D. D., Priel E., Yassur Y. (1995). Non-retinovascular leakage in diabetic maculopathy. *The British Journal of Ophthalmology*.

[16] Spaide R. F., Yannuzzi L. A., Marmor M. F., Wolfensberger T. J. (1998). Manifestations and pathophysiology of serous detachment of the retinal pigment epithelium and retina. *The Retinal Pigment Epithelium*.

[17] Ichiyama Y., Sawada O., Mori T., Fujikawa M., Kawamura H., Ohji M. (2016). The effectiveness of vitrectomy for diffuse diabetic macular edema may depend on its preoperative optical coherence tomography pattern. *Graefe's Archive for Clinical and Experimental Ophthalmology*.

[18] Korobelnik J. F., Lu C., Katz T. A. (2019). Effect of baseline subretinal fluid on treatment outcomes in VIVID-DME and VISTA-DME studies. *Ophthalmology Retina*.

[19] Moon B. G., Lee J. Y., Yu H. G. (2016). Efficacy and safety of a dexamethasone implant in patients with diabetic macular edema at tertiary centers in Korea. *Journal of Ophthalmology*.

[20] Das R., Spence G., Hogg R. E., Stevenson M., Chakravarthy U. (2018). Disorganization of inner retina and outer retinal morphology in diabetic macular edema. *JAMA Ophthalmology*.

[21] Murakami T., Nishijima K., Akagi T. (2012). optical coherence tomographic reflectivity of photoreceptors beneath cystoid spaces in diabetic macular edema. *Investigative Ophthalmology & Visual Science*.

[22] Liang M. C., Vora R. A., Duker J. S., Reichel E. (2013). Solid-appearing retinal cysts in diabetic macular edema: a novel optical coherence tomography finding. *Retinal Cases & Brief Reports*.

[23] Al Faran A., Mousa A., Al Shamsi H., Al Gaeed A., Ghazi N. G. (2014). Spectral domain optical coherence tomography predictors of visual outcome in diabetic cystoid macular edema after bevacizumab injection. *Retina*.

[24] Vujosevic S., Bini S., Torresin T. (2017). Hyperreflective retinal spots in normal and diabetic eyes: B-scan and en face spectral domain optical coherence tomography evaluation. *Retina*.

[25] Nishijima K., Murakami T., Hirashima T. (2014). Hyperreflective foci in outer retina predictive of photoreceptor damage and poor vision after vitrectomy for diabetic macular edema. *Retina*.

[26] Bolz M., Schmidt-Erfurth U., Deak G., Mylonas G., Kriechbaum K., Scholda C. (2009). Optical coherence tomographic hyperreflective foci: a morphologic sign of lipid extravasation in diabetic macular edema. *Ophthalmology*.

[27] Murakami T., Yoshimura N. (2013). Structural changes in individual retinal layers in diabetic macular edema. *Journal Diabetes Research*.

[28] Li F., Zhang L., Wang Y. (2018). One-year outcome of conbercept therapy for diabetic macular edema. *Current Eye Research*.

[29] Xu Y., Rong A., Xu W., Niu Y., Wang Z. (2017). Comparison of 12-month therapeutic effect of conbercept and ranibizumab for diabetic macular edema: a real-life clinical practice study. *BMC Ophthalmology*.

[30] Zhou Y., Wang C., Shi K., Yin X. (2018). Relationship between dyslipidemia and diabetic retinopathy: a systematic review and meta-analysis. *Medicine*.

[31] Shin Y. U., Hong E. H., Lim H. W., Kang M. H., Seong M., Cho H. (2017). Quantitative evaluation of hard exudates in diabetic macular edema after short-term intravitreal triamcinolone, dexamethasone implant or bevacizumab injections. *BMC Ophthalmology*.

[32] Sasaki M., Kawasaki R., Noonan J. E. (2013). Quantitative measurement of hard exudates in patients with diabetes and their associations with serum lipid levels. *Investigative Ophthalmology & Visual Science*.

[33] Joltikov K. A., Sesi C. A., de Castro V. M. (2018). Disorganization of retinal inner layers (DRIL) and neuroretinal dysfunction in early diabetic retinopathy. *Investigative Ophthalmology & Visual Science*.

[34] Nadri G., Saxena S., Stefanickova J. (2019). Disorganization of retinal inner layers correlates with ellipsoid zone disruption and retinal nerve fiber layer thinning in diabetic retinopathy. *Journal of Diabetes and its Complications*.

[35] Ghassemi F., Bazvand F., Roohipoor R., Yaseri M., Hassanpoor N., Zarei M. (2016). Outcomes of vitrectomy, membranectomy and internal limiting membrane peeling in patients with refractory diabetic macular edema and non-tractional epiretinal membrane. *Journal of Current Ophthalmology*.

[36] Zur D., Iglicki M., Busch C., Invernizzi A., Mariussi M. (2018). OCT biomarkers as functional outcome predictors in diabetic macular edema treated with dexamethasone implant. *Ophthalmology*.

[37] Ota M., Nishijima K., Sakamoto A. (2010). Optical coherence tomographic evaluation of foveal hard exudates in patients with diabetic maculopathy accompanying macular detachment. *Ophthalmology*.

[38] Ozkaya A., Alkin Z., Karakucuk Y. (2017). Thickness of the retinal photoreceptor outer segment layer in healthy volunteers and in patients with diabetes mellitus without retinopathy, diabetic retinopathy, or diabetic macular edema. *Saudi Journal of Ophthalmology*.

[39] Forooghian F., Stetson P. F., Meyer S. A. (2010). Relationship between photoreceptor outer segment length and visual acuity in diabetic macular edema. *Retina*.

[40] Endo H., Kase S., Takahashi M. (2020). Relationship between diabetic macular edema and choroidal layer thickness. *PLoS One*.

[41] Okamoto M., Matsuura T., Ogata N. (2016). Effects of panretinal photocoagulation on choroidal thickness and choroidal blood flow in patients with severe nonproliferative diabetic retinopathy. *Retina*.

[42] Rayess N., Rahimy E., Ying G. S. (2015). Baseline choroidal thickness as a predictor for response to anti-vascular endothelial growth factor therapy in diabetic macular edema. *American Journal of Ophthalmology*.

[43] Sonoda S., Sakamoto T., Yamashita T. (2014). Effect of intravitreal triamcinolone acetonide or bevacizumab on choroidal thickness in eyes with diabetic macular edema. *Investigative Ophthalmology & Visual Science*.

[44] Querques G., Lattanzio R., Querques L. (2012). Enhanced depth imaging optical coherence tomography in type 2 diabetes. *Investigative Ophthalmology & Visual Science*.

[45] Adhi M., Brewer E., Waheed N. K., Duker J. S. (2013). Analysis of morphological features and vascular layers of choroid in diabetic retinopathy using spectral-domain optical coherence tomography. *JAMA Ophthalmology*.

[46] Unsal E., Eltutar K., Zirtiloglu S., Dincer N., Ozdogan Erkul S., Gungel H. (2014). Choroidal thickness in patients with diabetic retinopathy. *Clinical Ophthalmology*.

[47] Eliwa T. F., Hegazy O. S., Mahmoud S. S., Almaamon T. (2017). Choroidal thickness change in patients with diabetic macular edema. *Ophthalmic Surgery, Lasers & Imaging Retina*.

[48] Kim J. T., Lee D. H., Joe S. G., Kim J. G., Yoon Y. H. (2013). Changes in choroidal thickness in relation to the severity of retinopathy and macular edema in type 2 diabetic patients. *Investigative Ophthalmology & Visual Science*.

[49] Okamoto M., Yamashita M., Ogata N. (2018). Effects of intravitreal injection of ranibizumab on choroidal structure and blood flow in eyes with diabetic macular edema. *Graefe's Archive for Clinical and Experimental Ophthalmology*.

[50] Lee H. K., Lim J. W., Shin M. C. (2013). Comparison of choroidal thickness in patients with diabetes by spectral-domain optical coherence tomography. *Korean Journal of Ophthalmology*.

[51] Rewbury R., Want A., Varughese R., Chong V. (2016). Subfoveal choroidal thickness in patients with diabetic retinopathy and diabetic macular oedema. *Eye*.

[52] Kim M., Ha M. J., Choi S. Y., Park Y.-H. (2018). Choroidal vascularity index in type-2 diabetes analyzed by swept-source optical coherence tomography. *Scientific Reports*.

[53] Sala-Puigdollers A., Figueras-Roca M., Hereu M. (2018). Repeatability and reproducibility of retinal and choroidal thickness measurements in diabetic macular edema using swept-source optical coherence tomography. *PLoS One*.

[54] Abadia B., Suñen I., Calvo P., Bartol F., Verdes G., Ferreras A. (2018). Choroidal thickness measured using swept-source optical coherence tomography is reduced in patients with type 2 diabetes. *PLoS One*.

[55] Iovino C., Pellegrini M., Bernabei F. (2020). Choroidal vascularity index: an in-depth analysis of this novel optical coherence tomography parameter. *Journal of Clinical Medicine*.

[56] Agrawal R., Gupta P., Tan K.-A., Cheung C. M. G., Wong T.-Y., Cheng C.-Y. (2016). Choroidal vascularity index as a measure of vascular status of the choroid: measurements in healthy eyes from a population-based study. *Scientific Reports*.

[57] Gupta C., Tan R., Mishra C. (2018). Choroidal structural analysis in eyes with diabetic retinopathy and diabetic macular edema-a novel OCT based imaging biomarker. *PLoS One*.

[58] Roy R., Saurabh K., Shah D., Chowdhury M., Goel S. (2019). Choroidal hyperreflective foci: a novel spectral domain optical coherence tomography biomarker in eyes with diabetic macular edema. *Asia-Pacific Journal of Ophthalmology*.

[59] AttaAllah H. R., Mohamed A. A. M., Ali M. A. (2019). Macular vessels density in diabetic retinopathy: quantitative assessment using optical coherence tomography angiography. *International Ophthalmology*.

[60] Parravano M., De Geronimo D., Scarinci F. (2019). Progression of diabetic microaneurysms according to the internal reflectivity on structural optical coherence tomography and visibility on optical coherence tomography angiography. *American Journal of Ophthalmology*.

[61] Veiby N. C. B. B., Simeunovic A., Heier M. (2020). Associations between macular OCT angiography and nonproliferative diabetic retinopathy in young patients with type 1 diabetes mellitus. *Journal Diabetes Research*.

